# Colorectal Cancer Screening in Hereditary and Familial High-Risk Populations: Best Practices and Future Directions

**DOI:** 10.1002/ijc.70615

**Published:** 2026-07-09

**Authors:** Ophir Gilad, Francesc Balaguer, Elizabeth E. Half, Kevin J. Monahan, Elena M. Stoffel, Sonia S. Kupfer

**Affiliations:** 1 Section of Gastroenterology, Hepatology and Nutrition, Department of Medicine, University of Chicago, Chicago, Illinois, USA; 2 Department of Gastroenterology, Clínic Barcelona, Centro de Investigación Biomédica en Red en Enfermedades Hepáticas y Digestivas, Institut D'investigacions Biomèdiques August Pi i Sunyer, Universitat de Barcelona, Barcelona, Spain; 3 Department of Gastroenterology, Rambam Health Care Campus, and the Bruce and Ruth Rappaport Faculty of Medicine, Technion Institute of Technology, Haifa, Israel; 4 The St Mark's Centre for Familial Intestinal Cancer, The National Bowel Hospital, Central Middlesex Hospital Site, London, UK; 5 Imperial College, London, UK; 6 Division of Gastroenterology and Hepatology, Department of Medicine, University of Michigan, Ann Arbor, Michigan, USA

**Keywords:** CRC screening, familial colorectal cancer, hereditary cancer syndromes

## Abstract

Colorectal cancer (CRC) remains a leading cause of cancer-related morbidity and mortality worldwide yet is largely preventable through effective screening and surveillance. While most CRC cases are sporadic, a substantial proportion occur in individuals at increased risk due to hereditary cancer syndromes or family history who require tailored screening strategies different from population-based approaches with respect to age of initiation, surveillance intervals, and modality. This review summarizes current evidence on CRC risk across higher risk groups, including Lynch syndrome, polyposis syndromes, carriers of moderate-penetrance genes, and individuals with a family history of CRC. Efficacy of colonoscopic surveillance and the potential roles of emerging biomarker tests and artificial intelligence-assisted technologies for detection of colorectal neoplasia are discussed. Current CRC surveillance guidelines, quality metrics and adherence in higher risk groups are reviewed. As research in genomics, biomarkers, microbiome, and artificial intelligence evolves, personalized risk-based screening strategies hold promise for optimizing CRC prevention. High-quality, population-specific data will be essential to refine surveillance intensity, improve adherence, and reduce CRC burden in higher risk populations.

## 1 ∣ Introduction

Colorectal cancer (CRC) is the third most common malignancy and cause of cancer-related deaths globally [1]. CRC can be detected early through screening that identifies early-stage cancers and might prevent cancer by removal of premalignant polyps. While most cases are sporadic [2, 3], identifying individuals at higher risk than the general population is essential, as they may require earlier or intensified screening, benefit from specific modalities, and warrant genetic testing. Higher risk groups include carriers of pathogenic variants in cancer predisposition genes, individuals with a family history of CRC, patients with long-standing inflammatory bowel disease, and those with a personal history of advanced colorectal neoplasia [3-5].

This review focuses on CRC risk and screening in individuals with hereditary or familial predisposition, while IBD and post-neoplasia surveillance are beyond the scope of this review and have been reviewed previously [6, 7].

## 2 ∣ Individuals at Higher Risk Compared to the General Population

Approximately 5%–10% of patients with CRC are found to carry a pathogenic genetic variant (PGV) in a cancer predisposition gene [8, 9]. Highly penetrant hereditary syndromes, such as Lynch syndrome and several polyposis syndromes, substantially increase CRC risk compared to the general population, whereas moderate-penetrance genes confer more modest risk elevations. Even in the absence of an identifiable genetic condition, a family history of CRC increases risk in proportion to the number of affected relatives.

### 2.1 ∣ Family History of CRC

Familial CRC, in which CRC occurs in individuals with affected relatives but no identifiable PGVs, accounts for about 25% of all CRC cases [10]. About 3%–10% of individuals have a first-degree relative (FDR) with CRC [10, 11]. Having an affected FDR confers a relative risk of approximately 2.25, increasing to 3.87 if the FDR had early-onset CRC (< 50 years), and to > 4 when multiple FDRs are affected [12, 13]. In contrast, second- and third-degree relatives confer substantially lower risk [14]. A family history of polyps also increases CRC risk (OR: 1.62), particularly when polyps are advanced, villous, diagnosed at younger ages, or present in multiple FDRs (OR: 1.70–1.8) [15].

### 2.2 ∣ Lynch Syndrome (LS)

The most common CRC predisposition syndrome with population prevalence of 1 in 279 individuals who carry a PGV in one of the DNA mismatch repair (MMR) genes [16]. LS accounts for about 2%–5% of CRC overall [17] and is also associated with increased risk of endometrial, gastric, ovarian, urothelial, biliary, pancreatic, and brain cancers [18]. LS is due to five genes associated with distinct cancer risk. PGVs in *MLH1, MSH2*, and *EPCAM* are the most highly penetrant, with lifetime CRC risks estimated at 30%–60%, whereas *MSH6* and *PMS2* are associated with lower CRC risks of 15%–30% and 9%–30%, respectively [19, 20]. Carriers of higher-penetrance genes also have increased risk of advanced adenomas, an independent risk factor for postcolonoscopy CRC [21]. In the general population, PGVs in *MLH1* and *MSH2* are less prevalent (1:1946 and 1:2841) than in *MSH6* and *PMS2* (1:758 and 1:714) [16].

### 2.3 ∣ Polyposis Syndromes

These syndromes can be broadly categorized based on polyp histology: adenomas, hamartomas, and serrated polyps.

The two best characterized adenomatous polyposis syndromes are familial adenomatous polyposis (FAP) and *MUTYH*-associated polyposis (MAP). FAP is a rare autosomal dominant syndrome caused by PGVs in the *APC* gene, with a prevalence of 1:8300–1:37,600 live births [22], while MAP is an autosomal recessive condition due to PGVs in both copies of the *MUTYH* gene. The prevalence of carrying one copy of a PGV in *MUTYH* in the general population is about 1%–2% [16]. Both FAP and MAP account for less than 1% of CRC cases [23]. Classic FAP is characterized by hundreds to thousands of polyps and a lifetime risk of CRC nearing 100% without colectomy, while attenuated FAP and MAP typically present with attenuated polyposis (< 100 adenomas) [24, 25]. Unscreened individuals with attenuated FAP or MAP have a lifetime CRC risk of up to 69%–75% [24, 26]. Rarer adenomatous polyposis syndromes include individuals with PGVs in *POLE*, *POLD1*, *AXIN2, NTHL1, MBD4*, and *MSH3*, collectively accounting for < 0.1% of CRC cases [2].

Hamartomas are abnormal growth of normal native tissue. Hamartomatous polyposis syndromes, including Peutz-Jeghers (PJS), juvenile polyposis (PJS), and *PTEN* hamartoma tumor syndrome (PHTS), are characterized by multiple gastrointestinal hamartomas as well as colonic and extracolonic cancers. PJS, caused by PGVs in the *STK11* gene, frequently presents with GI bleeding and/or intussusception due to small bowel polyps as well as characteristic mucocutaneous freckling. PJS incidence is estimated at 1 in 50,000–200,000 births, with a lifetime CRC risk of approximately 39% [27]. JPS is caused by alterations in *SMAD4* or *BMPR1*A, with PGVs identified in approximately 40%–60% of clinically diagnosed cases [28]. JPS typically presents with rectal bleeding and has an incidence of 1 in 100,000–160,000 births and confers a CRC risk of up to 68% by age 60 [29, 30]. PHTS, resulting from PGVs in *PTEN*, has an incidence of 1 in 200,000 live births and is characterized by macrocephaly, mucocutaneous lesions, autism spectrum disorder and increased risk for breast and thyroid cancers, with a more modest CRC lifetime risk of approximately 16% [31]. Substantial phenotypic overlap exists between the various polyposis syndromes, and multigene panel testing plays an important role in accurately identifying the underlying genetic diagnosis [32].

*Serrated polyposis syndrome* is defined as either ≥ 20 serrated lesions distributed throughout the colon with ≥ 5 proximal to the rectum, or ≥ 5 proximal serrated lesions measuring ≥ 5 mm, including at least two ≥ 10 mm, and is associated with an estimated CRC risk of approximately 20% [33]. Prevalence ranges from 0.01% to 0.05% in colonoscopy screening programs and diagnosis is primarily clinical, as most patients do not harbor identifiable PGVs in known polyposis gene [34].

### 2.4 ∣ Moderate Penetrance Genes

Individuals with moderate penetrance gene mutations have CRC risks intermediate between population risk and high-penetrance syndromes. PGVs in these genes are identified in up to 4.7% of CRC patients undergoing germline testing [35]. Examples include *CHEK2*, monoallelic *MUTYH* carriers, and the *APC* I1307K Ashkenazi Jewish founder variant [35]. CRC risk estimates for these genes vary. For example, *CHEK2* PGV carriers have demonstrated a modestly increased CRC risk in some populations (standardized incidence ratio 1.43 in the Netherlands) [36], while other large studies found no association [37]. Monoallelic *MUTYH* carriers appear to have a modestly elevated CRC risk in meta-analyses (OR: 1.16) [38, 39], which seems to be limited to specific variants [38], with low estimated lifetime CRC risks (6.39% for men and 4.42% for women), and an older median age at diagnosis of 67 years [40]. Accordingly, recent guidelines have shifted toward recommending that many of these individuals follow average-risk CRC screening recommendations [41, 42].

## 3 ∣ Age to Commence Screening and Surveillance Intervals

Surveillance recommendations for individuals at increased CRC risk vary according to risk estimates and age of onset. Higher cancer risk and earlier onset warrant earlier initiation of surveillance and more frequent screening intervals (Table 1).

### 3.1 ∣ Family History

For individuals with family history of CRC but without a known hereditary syndrome, multiple guidelines recommend initiating screening at age 40 or 10 years before the age at diagnosis of the youngest affected FDR [3, 47]. The U.S. Multi-Society Task Force recommends 5-year surveillance intervals for those with an FDR diagnosed < 60 or with two affected FDRs, and 10-year intervals if the FDR was diagnosed ≥ 60 [48]. However, data from the prostate, lung, colorectal and ovarian cancer (PLCO) trial showed no significant risk difference by age at diagnosis of the affected relative [49]. Although randomized trials comparing earlier versus standard screening are lacking, modeling studies indicate that earlier initiation and shorter surveillance intervals are cost-effective, particularly with multiple affected FDRs, and support extending intervals after negative colonoscopy beyond age 50 [50]. Some European guidelines, where national screening programs are largely stool-tests based, recommend intensified colonoscopy for individuals meeting Amsterdam criteria (≥ 3 FDRs with CRC across ≥ 2 generation, with one a FDR of the other two) while those not meeting these criteria are recommended to undergo a one-time colonoscopy at age 55, with further surveillance only if polyps are found [4]. A subset of families fulfilling Amsterdam criteria without an identifiable PGV are classified as familial CRC type X syndrome and are recommended to undergo colonoscopy every 3 years [51].

### 3.2 ∣ Lynch Syndrome

Until recently, LS guidelines did not differentiate surveillance recommendations by gene. As it became evident that LS has unique genotypic risks, guidelines stratify surveillance recommendations based on gene. Although recommendations vary slightly across gastrointestinal and oncology societies, the overarching principles are consistent: carriers of *MLH1*, *MSH2*, and *EPCAM*, the higher-penetrance genes, are advised to begin colonoscopy surveillance in their 20s with more frequent intervals, whereas *MSH6* and *PMS2* carriers typically initiate screening in their 30s with less frequent examinations [4, 5, 41, 43].

### 3.3 ∣ Polyposis Syndromes

In FAP, guidelines recommend initiating colonoscopy surveillance at ages 10–15 and repeating at 1–2-year intervals. Surveillance continues until polyp burden becomes unmanageable, at which point colectomy is recommended, with the choice of procedure guided by rectal polyp burden [4, 41, 44, 45]. Postoperative surveillance of the ileal pouch or retained rectum is advised every 6–12 months [4, 41, 44, 45], with some guidelines recommending shorter intervals of 3–6 months for high-risk polyps and longer intervals up to 2 years when no high-risk lesions are present [52]. An emerging alternative strategy, first explored in Japan, is intensive downstaging polypectomy, which involves frequent colonoscopies with sequential removal of larger polyps, followed by lengthening intervals once polyp burden is controlled. In a multicenter Japanese study, 88% of patients avoided colectomy over 5 years [53]. However, long-term outcomes are unknown, and this approach is not currently endorsed by gastrointestinal or oncology societies.

In attenuated FAP and MAP, polyp burden is generally lower and onset later than in classic FAP. Accordingly, guidelines recommend initiating colonoscopy surveillance in the late teenage years and no later than 25–30, with repeat examinations every 1–2 years. Prophylactic colectomy is typically unnecessary but may be considered when endoscopic control becomes unfeasible [4, 41, 44, 45].

For hamartomatous polyposis syndromes, including PJS and JPS, colonoscopic surveillance is recommended every 1–3 years starting in early adolescence [4, 41, 45]. In PHTS, given the lower CRC risk, screening typically begins at age 35 and is repeated every 5 years or more frequently based on polyp burden [41].

In serrated polyposis syndrome, guidelines recommend colonoscopic surveillance every 1–3 years from the time of diagnosis [4, 41, 45]. Surveillance outcomes appear favorable: a Dutch prospective study of 142 patients followed for 10 years identified only one CRC, corresponding to a 5-year cumulative incidence of 1.0% [54]. Retrospective studies have reported similarly low CRC incidences (0%–1.9%) [55, 56]. Achieving control of polyp burden typically requires two to three colonoscopies, after which surveillance intervals may be safely extended [54, 55].

### 3.4 ∣ Moderate Penetrance Genes

For carriers of moderate-penetrance genes, guidelines have increasingly shifted away from intensified surveillance, given the relatively modest increase in CRC risk compared with the general population. Carriers of *CHEK2* pathogenic variants or monoallelic *MUTYH* variants are now generally advised to follow population-based screening recommendations, modified by personal or family history [41]. In contrast, some guidelines continue to recommend colonoscopy beginning at age 40 and repeated every 5 years for carriers of the *APC* I1307K variant [41]. A position statement by the International Society for Gastrointestinal Hereditary Tumors recommends intensified surveillance only for carriers of Ashkenazi Jewish ancestry, based on evidence demonstrating an increased CRC risk in this population (OR: 1.68), with no convincing evidence of elevated risk in non-Jewish populations [42, 57]. Notably, available data do not support earlier CRC onset in *APC* I1307K carriers, and surveillance intervals of 5 years beginning at standard screening age were therefore recommended [42].

## 4 ∣ Efficacy of CRC Screening in High-Risk Individuals

Longer term, prospective data have shown efficacy of CRC screening in individuals with family history of CRC and LS, while for more rare conditions, high-quality data are sparse. Table 2 summarizes available studies on CRC screening efficacy in higher risk groups.

### 4.1 ∣ Family History

Efficacy of colonoscopy in individuals with a family history of CRC was demonstrated in a 16-year prospective study of 1678 high-risk individuals, including those with one to three FDRs (moderate risk) and those meeting Amsterdam criteria (high-risk). Compared with expected rates, CRC incidence was reduced by 92% in the moderate-risk group and 51% in the high-risk group, with a corresponding mortality reduction of 81% and 72% [69]. A modeling study similarly estimated that colonoscopy could prevent 66%–95% of CRC cases and 91%–97% of CRC deaths and suggested that a 10-year colonoscopy interval with biennial fecal immunochemical test (FIT) achieves comparable efficacy while reducing the number of colonoscopies and overall costs [70].

### 4.2 ∣ Lynch Syndrome

The effectiveness and optimal interval of endoscopic surveillance in LS have been evaluated in several studies. An early landmark study, comparing 133 LS carriers undergoing colonoscopy every 3 years with 119 unscreened controls, demonstrated a 62% reduction in CRC incidence and no CRC-related deaths in the surveillance group, compared with nine deaths among controls [58]. A subsequent single-center study reported that increased adenoma detection and shorter surveillance intervals were associated with cumulative CRC risk reductions ranging from 13% to 29% [59]. Cost-effectiveness models similarly suggest that carriers of higher-penetrance genes derive greater benefit from earlier and more frequent surveillance than those with lower-penetrance genes [60].

However, data from multinational, prospective studies have yielded more nuanced results. A multinational study of 2474 LS patients managed with different surveillance intervals (every year, every 1–2 years, or every 2–3 years) found no significant difference in CRC incidence between intervals [61]. Additionally, a large multicenter Spanish study reported lower CRC incidence among individuals undergoing colonoscopy at intervals of ≤ 3 years compared with > 3 years; however, no difference was observed when comparing surveillance intervals of ≤ 2 years versus > 2 years [62]. Data from the Prospective Lynch Syndrome Database (PLSD) further showed that 58% of CRCs occurred despite adherence to a 2-year surveillance interval, and 79% despite adherence to a 3-year interval, raising questions about the extent to which colonoscopy reduces CRC incidence in LS [63]. Notably, this cohort demonstrated excellent post-diagnosis survival, with a 10-year survival rate of 91%. These findings reflect outcomes over more than two decades of surveillance, during which endoscopic technology has evolved. Moreover, colonoscopy quality metrics were not systematically reported, suggesting that these results may represent a conservative or “worst-case” estimate of surveillance effectiveness [63].

### 4.3 ∣ Polyposis Syndromes

In FAP, although no randomized controlled trials have compared effectiveness of screening, multiple observational studies have demonstrated a reduction in CRC incidence in patients undergoing surveillance. A large systematic review that included 30 studies that compared CRC incidence in screened vs. symptomatic FAP patients showed a statistically significant reduction in CRC incidence with screening (OR: 0.01–0.37), as well as significant reduction in CRC-related mortality (OR: 0.01–0.16) [64]. These findings are supported by population-based studies from Denmark and the Netherlands, which demonstrated substantially higher 10-year survival (0.97 vs. 0.42, *p* < 0.001), and lower incidence of CRC (0.04 vs. 0.47) among screened relatives compared with probands [65, 66]. Collectively, these data suggest that colonoscopic surveillance facilitates timely colectomy before the development of CRC in patients with FAP.

Outcome data for hamartomatous syndromes are limited. A Danish population-based study of 56 PJS patients reported four CRC cases, only one of which was detected during routine surveillance [67]. Similarly, a Scottish population-based study of 28 JPS patients found that none of seven CRCs occurred during surveillance [68]. Although population-based, these studies are constrained by small sample sizes, limiting definitive conclusions regarding surveillance effectiveness.

## 5 ∣ Screening Modalities

**FIGURE 1 ∣:**
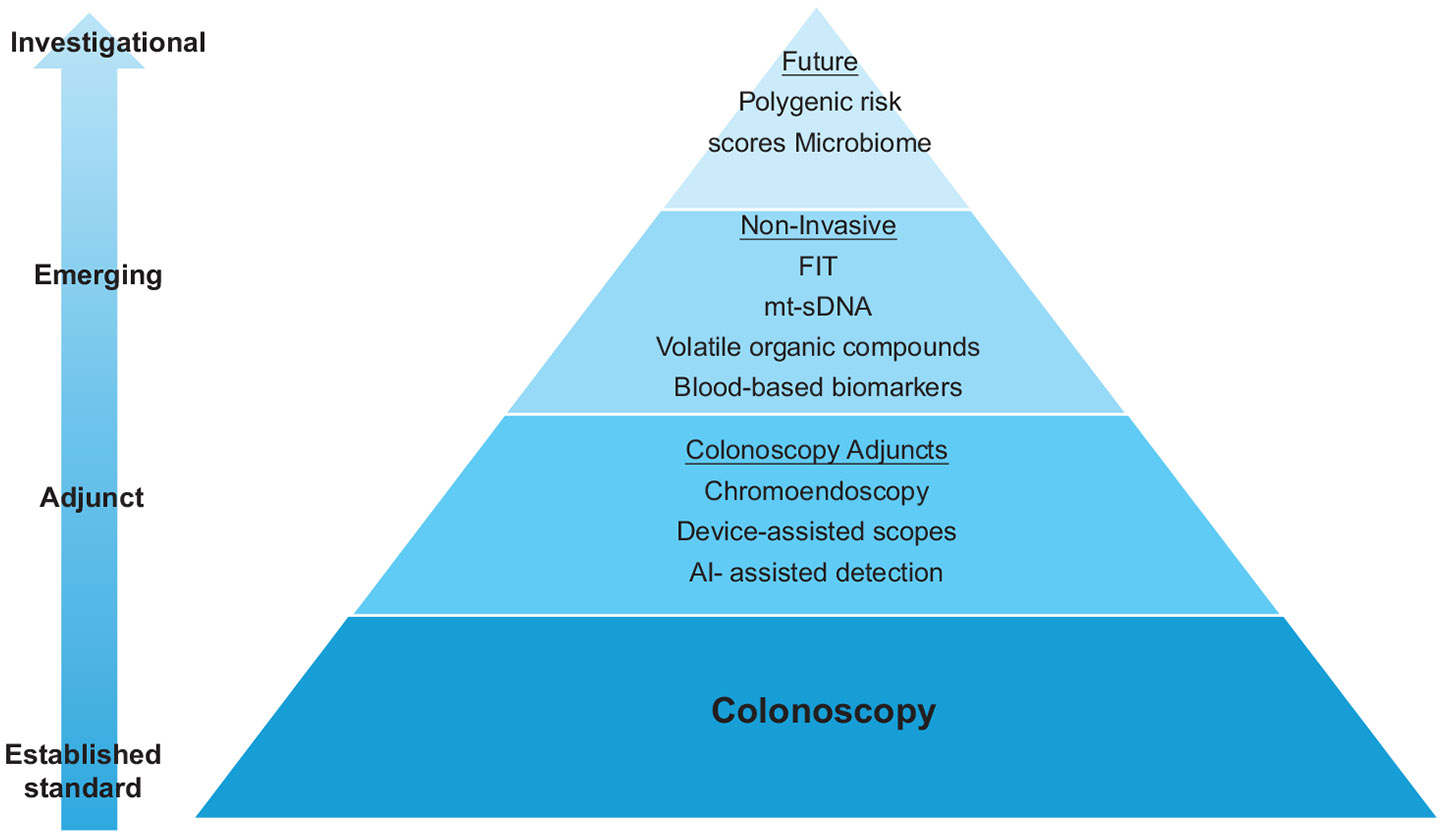
Conceptual hierarchy of colorectal cancer screening modalities in high-risk populations. Colonoscopy remains the cornerstone of screening, enabling prevention and early detection. Adjunct endoscopic techniques may enhance lesion detection but have limited outcome data. Noninvasive modalities and blood-based biomarkers remain investigational and are currently evaluated primarily as adjuncts rather than replacements for colonoscopy.

While both colonoscopy and noninvasive modalities are acceptable first-line options in average-risk populations, in high-risk individuals, noninvasive approaches have not been adequately evaluated and several studies are currently underway (Figure 1).

### 5.1 ∣ Colonoscopic Screening

Colonoscopy remains the cornerstone of CRC screening in high-risk individuals. In LS, colonoscopy enables early detection and removal of premalignant polyps, especially in the right colon. In polyposis syndromes, colonoscopy is essential for assessing polyp burden, guiding decisions regarding prophylactic surgery, and, in selected cases, downstaging disease.

The role of chromoendoscopy in LS remains controversial. Although early back-to-back studies demonstrated increased adenoma detection rates (ADR) [71, 72], larger studies and meta-analyses showed similar ADR with white-light endoscopy (28%) and chromoendoscopy (31%) [73-75]. Data in polyposis syndromes are limited, and additional lesions detected in FAP were generally diminutive [76, 77]. Distal attachment devices to enhance mucosal visualization improve ADR in the general population [78], and may improve detection of serrated polyps and right-sided lesions [79, 80], but data in high-risk populations remain scarce. Artificial intelligence (AI)-assisted colonoscopy increases ADR in average-risk populations, particularly for small, nonadvanced lesions and among less experienced endoscopists [81, 82]. However, studies in LS have not demonstrated significant benefit, including a large randomized trial showing no difference in ADR [83, 84].

### 5.2 ∣ Nonendoscopic Screening Modalities

Stool-based tests offer convenient, noninvasive screening options but are not recommended for high-risk individuals. FIT has a sensitivity of approximately 79% for CRC and 25%–56% for advanced neoplasia, although performance is highly dependent on the cut-off used [85]. Multitarget stool DNA (mt-sDNA) testing demonstrates higher sensitivity for CRC (~94%) but remains limited for advanced neoplasia (~43%) [86]. These tests have not been validated in high-risk populations, and their limited sensitivity for precursor lesions, which may progress rapidly in these individuals, precludes their routine use [87].

During the COVID-19 pandemic, FIT was temporarily used for LS stratification in the United Kingdom. At a cutoff ≥ 10 μg/g, FIT had 64.7% sensitivity, 66% specificity, 23.4% positive predictive value (PPV), and 92.1% negative predictive value (NPV) [88]. In a prospective Dutch study of 217 LS patients, cutoffs of < 4.1 μg/g demonstrated high sensitivity for CRC and advanced adenoma (89%) as well as high NPV (99%) [89]. FIT as an adjunct to colonoscopy in LS patients is currently being evaluated in both the United Kingdom [90] and the United States (NCT06898996).

The efficacy of mt-sDNA test in high-risk individuals was evaluated in a large retrospective study and was noted to have similar PPV to that of the average risk population. However, the high-risk cohort was heterogenous and included individuals with personal history of colorectal neoplasia, aerodigestive malignancies, inflammatory bowel disease, as well as patients with worrisome signs or symptoms such as rectal bleeding or iron deficiency anemia, for whom noninvasive testing is inappropriate [91]. The sensitivity and specificity of mt-sDNA test for colorectal neoplasia in LS is currently being evaluated in the CORAL study (NCT05410977).

Another noninvasive marker for colorectal neoplasia is the detection of volatile organic compounds using breath- or stool-based assays. In the general population, these compounds have demonstrated relatively high sensitivity and specificity for the detection of colorectal polyps of any risk category [92]. In LS, volatile organic compounds showed high sensitivity and NPV for CRC and advanced adenomas (89%–100%), with similar high sensitivity for any colorectal neoplasia (84%–88%), although specificity was more modest (28%–44%) [93].

Blood-based biomarkers represent the most recent addition to noninvasive CRC screening modalities. Cell-free DNA and methylation-based assays demonstrate sensitivities of 79%–83% for CRC detection; however, sensitivities are substantially lower for early-stage cancers (55%–57%) and advanced precursor lesions (12.5%–13.2%), and these assays have not been validated in high-risk populations [94]. A small study comparing 18 LS patients with endoscopic lesions to 18 without demonstrated the potential utility of blood-based microsatellite instability testing. This assay, which evaluates five microsatellite markers, yielded an area under the curve of 0.80, with sensitivity of 75%, specificity of 72%, PPV of 42%, and NPV of 93% [95].

Computed tomographic (CT) colonography is an alternative for CRC detection, demonstrating good sensitivity (90%) and specificity (86%) for lesions ≥ 10 mm [96]. A study in individuals with a family history of CRC reported similar performance (sensitivity 82%, specificity 93%) [97]. However, no studies have evaluated CT colonography in hereditary cancer syndromes.

## 6 ∣ Quality Metrics

In the general population, colonoscopy is operator dependent, and higher ADRs are associated with lower rates of interval CRC [98]. High-quality screening colonoscopy relies on adequate bowel preparation, high cecal intubation rates, sufficient withdrawal time, and appropriate adenoma and sessile serrated lesion detection rates, as well as proper documentation, appropriate procedural indication, correct polyp resection techniques, and adherence to recommended surveillance intervals [99]. However, quality metrics in high-risk populations are less well defined.

In LS, a large multicenter Spanish study demonstrated that adequate bowel preparation and complete colonoscopy were associated with higher ADR; however, there was only a non-significant trend toward fewer interval cancers associated with these metrics [62]. A Dutch registry study reported that 9% of interval cancers arose in a previously unexamined portion of the colon, underscoring the importance of complete colonoscopy [100]. A French LS network implemented a structured quality-improvement protocol in which LS carriers with inadequate bowel preparation repeated colonoscopy within 3 months; those without chromoendoscopy or with adenomas repeated colonoscopy within 1 year; and all others underwent repeat colonoscopy every 2 years. Comparison of outcomes before and after implementation demonstrated a lower CRC incidence (2.8% vs. 0.3%, *p* = 0.012) without a significant change in ADR. Notably, the post-implementation period was associated with a higher detection rate of flat adenomas (15.5% vs. 6.1%, *p* < 0.005) [101].

While the general population ADR benchmark is ≥ 35% [99], no optimal threshold has been defined in LS, where reported ADRs range from 10%–25% [62, 102, 103], and no study has linked a specific target to improved long-term outcomes. No data specifically addresses quality metrics in polyposis syndromes, moderate-penetrance gene carriers, or individuals with a family history of CRC, and current guidelines therefore apply general population quality standards to these groups [4].

## 7 ∣ Adherence

Adherence to surveillance is critical for CRC prevention, particularly in high-risk individuals, as failure to undergo recommended surveillance represents a missed opportunity to remove precancerous lesions and detect cancers at an early stage. Long-term adherence to colonoscopy in LS is variable, ranging from 60% to 88% [104]. In a survey of 197 LS carriers, 21% reported that colonoscopy significantly impacted their quality of life, and 57% described it as moderately to extremely burdensome; however, perceived burden was not associated with noncompliance, whereas a lower educational level was [105]. Similarly, an Italian survey study observed higher adherence among individuals with higher education, those who received genetic counseling, and those living closer to a referral center [106].

In FAP, adherence to colonoscopy surveillance is also variable. A small Saudi Arabian study reported that 78.5% of patients underwent annual colonoscopy prior to surgery, but only 38.4% completed surveillance colonoscopy 1 year postoperatively [107]. In contrast, a larger Swedish study reported post-surgical surveillance adherence in 86% of respondents [108]. Colonoscopy uptake among FDRs of patients with CRC is lower than that observed in individuals with hereditary cancer syndromes. In one study of 342 FDRs of CRC patients, only 38% agreed to undergo colonoscopy [109]. Another survey found that only 63% of CRC patients reported receiving a recommendation for their siblings to undergo colonoscopy; among these, 20.5% reported that all siblings underwent colonoscopy, 25.6% reported partial uptake, and 51.2% reported that no relatives underwent colonoscopy [110]. Factors associated with higher compliance among FDRs include younger age at CRC diagnosis in the affected relative, female sex, higher education level, stable employment, married status, urban residence, and receipt of a recommendation from a healthcare professional [111].

## 8 ∣ Future of CRC Screening in High-Risk Populations

The next step in CRC screening for high-risk populations is the personalization of surveillance strategies based on an individual's risk profile. Polygenic risk scores integrating genetic and clinical data are being evaluated to stratify risk and guide tailored screening [112]. Emerging blood- and stool-based biomarkers may further refine risk assessment, identifying individuals who require closer surveillance while allowing others to safely extend colonoscopy intervals [90, 95, 113]. The gut microbiome may provide an additional layer of risk stratification, as specific microbial signatures and metabolites have been associated with differential CRC risk [114-116].

Advances in endoscopic technology, including AI, are expected to improve detection of high-risk lesions. Beyond endoscopy, AI may enhance risk prediction using demographic, clinical, and laboratory data [117]. Although most tools are currently being developed and validated in average-risk populations, they are expected to have important applications in high-risk individuals, who may derive greater benefit from precision-based screening given their elevated baseline risk of CRC.

## 9 ∣ Summary

High-risk individuals represent a population in whom effective screening and surveillance can meaningfully reduce cancer morbidity and mortality. While colonoscopy remains the cornerstone of CRC prevention across high-risk conditions, optimal strategies vary considerably by underlying risk, gene penetrance, and phenotype, underscoring the importance of tailored surveillance recommendations. Existing evidence highlights the benefits and limitations of current screening paradigms and reveals persistent gaps in quality metrics, adherence, and validation of emerging technologies in these populations. As advances in genomics, biomarkers, microbiome science, and artificial intelligence continue to evolve, integrating these tools into personalized screening frameworks holds promise for improving cancer prevention. Future efforts should focus on generating high-quality, population-specific data to refine risk stratification, optimize surveillance intensity, and enhance long-term adherence in individuals at increased risk for CRC.

## Figures and Tables

**TABLE 1 ∣:** Colonoscopic surveillance recommendations across high-risk colorectal cancer populations.

High-risk group	Lifetime CRC risk estimates	Age to initiate surveillance	Surveillance interval	Comments
Lynch syndrome [4, 5, 41, 43]				
*MLH1*	40%–60%	20–25	1–2 years	European guidelines recommend initiating surveillance at older ages (25 or 35) and repeating every 2 years
*MSH2/EPCAM*	30%–50%	20–25	1–2 years	
*MSH6*	15%–30%	30–35	1–3 years	
*PMS2*	9%–30%	30–35	1–3 years	
Familial adenomatous polyposis [4, 41, 44, 45]				
Classic	~100%	10–15	1–2 years	Surveillance continues until polyp burden is unmanageable
Attenuated	70%	12–18	1–2 years	
Post colectomy	Rectal 10%–30%, pouch < 1%–3%	From time of surgery	6–12 months	
*MUTYH*-associated polyposis [4, 41, 44, 45]	75%	Late teenage years, no later than 25–30	1–2 years	Similar to attenuated FAP
Peutz Jeghers syndrome [4, 41, 45]	39%	8–10	1–3 years	
Juvenile polyposis syndrome [4, 41, 45]	68%	10–15	1–3 years	
*PTEN* hamartoma tumor syndrome [41]	16%	35	5 years	European guidelines recommend that if baseline colonoscopy is negative, patients should not undergo intensified CRC screening [46]
Serrated polyposis syndrome [4, 41, 45]	20%	From time of diagnosis	1–3 years	
Moderate penetrance genes				
*CHEK2* [41]	2.5%–7%	45	10 years	Intensified surveillance no longer routinely recommended. Time of first colonoscopy and interval modified by family or personal history of CRC
Monoallelic *MUTYH* [41]	6.4%	45	10 years	
*APC* I1307K				
Ashkenazi [41, 42]	5%–10%	40–45	5 years	NCCN guidelines do not recommend distinguishing between Ashkenazi and non-Ashkenazi carriers
Non-Ashkenazi [41, 42]		45	10 years	
Family history of CRC [3, 5, 47, 48]	9%–20%	40 or 10 years before youngest affected FDR	5–10 years	European guidelines recommend 5-year intervals. US guidelines advise 5-year intervals when FDR diagnosed before age 60, and 10-year interval otherwise. British guidelines recommend 5-year colonoscopy starting at age 40 only for individuals with 3 FDRs across 1 generation; those with 2 FDRs or 1 FDR diagnosed before age 50 are advised a one-time colonoscopy at age 55 and with further surveillance determined by polyp findings [4]

Abbreviations: CRC, colorectal cancer; FAP, familial adenomatous polyposis; FDR, first degree relative; NCCN, National Comprehensive Cancer Network.

**TABLE 2 ∣:** Efficacy of screening in high-risk conditions.

High-risk group	Primary role of colonoscopy	Risk mitigation evidence for colonoscopy	Risk mitigation evidence for noninvasive tests*
Lynch syndrome [58-63]	Possible prevention by removal of premalignant polyps and early detection	Early prospective study showed 56%–62% reduction in CRC incidence and elimination of CRC-related mortality; later observational and registry data show high rates of interval cancers and no clear incidence difference between 1-, 2-, and 3-year intervals	FIT used as triage (sensitivity 64.7%–89%, specificity 66%–28% depending on cutoff);VOCs sensitivity 89%–100% for CRC/advanced adenoma, specificity 28%–44%
Familial adenomatous polyposis [64-66]	Diagnosis of advanced lesions Assessment of polyp burden—guide timing of prophylactic surgery Post-surgical surveillance Disease downstaging (not routinely used)	Observational data show marked CRC reduction (OR: 0.01–0.37) and CRC mortality reduction (OR: 0.01–0.16); population-based studies demonstrate substantially improved 10-year survival (0.97 vs. 0.42) and lower CRC incidence in screened relatives vs. probands	No noninvasive studies available
Hamartomatous polyposis syndromes [67, 68]	Prevention and early detection	Limited data; small population-based studies show CRC cases occurring despite surveillance; no robust evidence demonstrating incidence reduction	No noninvasive studies available
Serrated polyposis syndrome [54-56]	Prevention and early detection	5-year CRC incidence 1% during a 10-year prospective surveillance period. Incidence of 0%–1.9% in retrospective studies	No noninvasive studies available
Family history of CRC [9, 69, 70]	Prevention and early detection	51%–92% reduction of CRC incidence and 72%–71% reduction in mortality in prospective study. Modeling predicts 66%–95% CRC prevention and 91%–97% mortality reduction.	FIT evaluated as adjunct in modeling (10-year colonoscopy + biennial FIT comparable efficacy); CT colonography sensitivity 82%, specificity 93% in family history cohort

Abbreviations: CRC, colorectal cancer; CT, computed tomography; FIT, fecal immunochemical test; VOC, volatile organic compounds.

* Noninvasive tests not validated for routine use.

## Abbreviations:

ADR: adenoma detection rate
AI: Artificial intelligence
CRC: colorectal cancer
FAP: familial adenomatous polyposis
FDR: first-degree relative
FIT: fecal immunochemical test
JPS: juvenile polyposis syndrome
LS: Lynch syndrome
MAP: *MUTYH*-associated polyposis
MMR: mismatch repair
mt-sDNA: multitarget stool DNA
PGV: pathogenic genetic variant
PHTS: *PTEN* hamartomatous tumor syndrome
PJS: Peutz-Jeghers syndrome
VOC: volatile organic compounds
